# Chemical components analysis of handmade alcoholic beverages with special focus on psychoactive drugs as adulterants

**DOI:** 10.1016/j.mex.2025.103230

**Published:** 2025-02-17

**Authors:** Morvarid Sadat Miri, Dariush Badakhshan, Maryam Akhgari, Masoud Ghadipasha

**Affiliations:** a Department of Toxicology & Pharmacology, Faculty of Pharmacy, Pharmaceutical Sciences Branch, Islamic Azad University (IAUPS), Tehran Province, District 1, Yakhchal Street, Yasaman Alley, Tehran 19395/1495, Iran; b Legal Medicine Research Center, Legal Medicine Organization, Behesht Street, South Side of the City Park, Misagh Alley, Tehran 1114795113, Iran

**Keywords:** Handmade alcoholic beverages, Forensic toxicology, Psychoactive drugs, Gas chromatography, Adulteration, Gas Chromatography and High Performance Liquid Chromatography

## Abstract

Handmade alcoholic beverages are produced in clandestine laboratories. There are reports concerning the of counterfeit alcoholic beverages adulterated with psychoactive drugs. The purpose of this study should be to analyze volatile components and psychotropic substances in handmade alcoholic beverages. In this study, 616 samples were analyzed for the detection of ethanol, methanol, isopropanol, and acetone using gas chromatography. High-performance liquid chromatography was used for the analysis of benzodiazepines, nicotine, morphine, codeine, methadone, tramadol, and methamphetamine. Results were analyzed statistically by SPSS 25 and EXCEL 16 software. These beverages are sold in Iranian drug black market without regulatory and market oversight with increased risk of safety issues. In conclusion, it is important to know the drug profile in alcoholic beverages to investigate the cause and manner of poisoning with these kinds of fake beverages.

• Ethanol was found in 86 % of the samples, while methanol, a toxic contaminant, was present in 8 %.
• Several psychotropic substances, including methadone, tramadol, morphine, codeine, nicotine, benzodiazepines, and methamphetamine, were identified, showing intentional adulteration. The most common adulterants were methadone (24 %), diazepam (16.1 %), and tramadol (13.8 %).
• Furthermore, 36.8 % of adulterated samples contained at least one benzodiazepine, reflecting widespread tampering in alcoholic beverages.

## Graphical abstract

**Figure:**
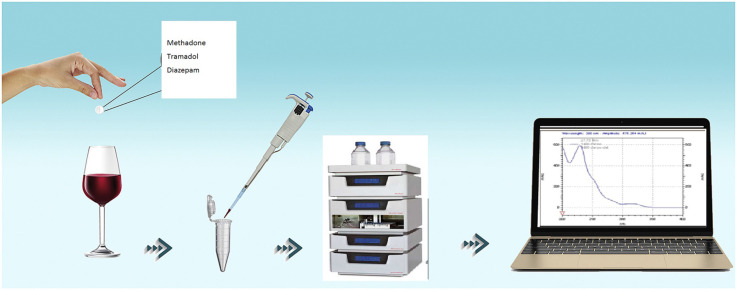

Specifications table

**Table:** 

Subject area:	Pharmacology, Toxicology and Pharmaceutical Science
More specific subject area:	Forensic Toxicology
Name of your method:	Gas Chromatography and High Performance Liquid Chromatography
Name and reference of original method:	Gas Chromatography and High Performance Liquid Chromatography that were set for the determination of alcohols and drug adulterants in alcoholic beverages
Resource availability:	None

## Background

Alcoholic beverages and alcohol are used interchangeably to refer to a product with various amounts of ethanol that is consumed worldwide as part of recreational or ceremonial activities [1]. According to World Health Organization (WHO) report in 2018, 280 million people in the world have alcohol use disorder [2]. Use of alcoholic beverages has been banned for Iranian citizens since 1979. The law is against production, selling, possession and consuming any kind of alcoholic beverages in Iran. It can be considered as criminal act according to religious and legal rules. Therefore, governors have faced an uphill battle to gather and fight against bootleg alcoholic beverages [3]. However, production and use of handmade and illegal alcoholic beverages is common in Iran due to limited access to standard alcoholic drinks [4]. This means that there is no regulation on the manufacturing and quality of alcohol containing beverages and much of them are produced in clandestine laboratories or home kitchens. Research from previous colleagues confirmed that some handmade and counterfeit alcoholic beverages were adulterated with active pharmaceutical ingredients (APIs) [5]. This issue encourages alcohol users to buy alcohol from dealers that produce alcohols with more psychological effects. Previous colleagues analysed handmade alcoholic beverages to detect volatile substances ethanol, methanol, isopropanol and acetone, but did not focus on APIs or other adulterants deliberately added to alcoholic drinks. Dadpour et al. in their study on noncommercial alcoholic beverages referred to Legal Medicine Organization, Mashhad, Iran, found ethanol and methanol in alcoholic beverages in different concentration ranges using gas chromatography instrumentation. However, they did not focus on other psychoactive, legal or illegal drugs [6]. The advantage of the present study is to analyse handmade alcoholic beverages for the detection of volatile compounds such as ethanol, methanol, isopropanol and acetone and also added psychotropic drugs to drinks simultaneously. In a case study, a man was arrested and a plastic bag containing alcoholic drink was detected in his house. Results of alcoholic beverage analysis using gas chromatography/mass spectrometry confirmed methamphetamine presence in alcoholic beverage [7]. ɣ-Butyrolactone and ɣ-hydroxybutyric acid were detected in beer, wine, rum and cola in the study of Davis et al. Analysed samples contained various additional compounds like fermentation by products and flavoring agents such as hexanoic acid, phenethyl alcohol, benzoic acid, caprylic acid, nonanoic acid, and glycerol [8]. Alprazolam, chloral hydrate and diazepam were detected using HPLC instrumentation in the study of Rao et al. [9]. We encountered methanol poisoning related deaths in many provinces in Iran during COVID-19 pandemic period [10]. Therefore, it is needed to analyse with new approaches to the problem to detect alcoholic beverages falsification and adulteration with APIs and other alcohols such as methanol.

With an annual production of 150,000 tons of grapes, the Markazi province, Iran is one of the important poles of the production of this product in the country [11]. Some of those grapes are used for the production of illegal alcoholic beverages and also adulteration may take place by dealers. Any kind of products that are in high demand by the young population and have a highly profitable market are gaining popularity for targeted adulteration and falsification [12]. As a matter of fact, adulterated alcoholic beverages represent a growing issue for public health. There are informal reports regarding hospital admission due to alcohol poisoning with unexpected and unpredictable clinical manifestations in Markazi Province, Iran, supposing that alcoholic beverages are adulterated with legal and illegal APIs.

In order to fill this gap of knowledge, we conducted the present study with the aim of analysis handmade alcoholic beverages for the qualitative and quantitative detection of volatile ingredients and added legal and illegal APIs in Markazi province, Iran. To the best of our knowledge this is the first work that had been conducted in Iran for handmade alcoholic beverage drug profiling using quantitative instrumental analysis.

## Equipment & materials

### HPLC instrumentation and analysis condition

Quantitative analysis of samples for the detection of psychotropic drugs was performed using high performance liquid chromatography (HPLC) instrumentation from KNAUER Company (Germany), equipped with photo diode array, PDA-2800 detector. Psychotropic drugs were separated on a Eurospher II 100 Å C-18 (100 mm × 3 mm), particle size 3 µm column obtained from KNAUER Company (Germany). To push the mobile phase through the column, two high-pressure pumps, with a degasser module and with a mixing chamber, were used. EZChrom chromatographic data system was used for data acquisition, integration, and processing. The mobile phase was a mixture of phosphate buffer adjusted to pH=2.3 and acetonitrile (65:35 v/v) with a flow rate of 1 mL/min and the pressure was about 100–120 MPa. Loop volume was 10 µL.

### GC instruments and analytical conditions

Alcohol concentrations were routinely determined using a quantitative gas chromatography (GC) (YL6500, Young Lin, Korea) equipped with a flame ionization detector (FID). The column was GC packed column (2.0 m, *L* × 1/8″ × 2.0 mm, ID), packed with Porapak Q, 80/100 mesh. Nitrogen (flow rate: 30 mL/min) was used as carrier gas. Detector gas was a mixture of hydrogen (produced by a hydrogen generator) and the air. Injector, detector and oven temperatures were set at 210 °C, 240 °C and 170 °C respectively. Identification and quantification of volatile analytes was accomplished using a flame ionization detector.

### Materials

Methanol, ethanol, isopropanol, acetone, acetonitrile, (HPLC grade solvents), chloroform, phosphoric acid, potassium dihydrogen phosphate (KH2PO4), boric acid, sodium tetraborate, hydrochloric acid, and sodium hydroxide were purchased from Merck Chemical Co. (Darmstadt, Germany). Buffers, mobile phase for HPLC system, and eluents were prepared with HPLC grade water for chromatography (Merck Millipore). HPLC grade solvents are high purity solvents with minimal UV-absorbing impurities distilled and filtered through 0.2-micron filters, manufactured especially for use with HPLC and UV spectroscopy.

Diazepam, oxazepam, flurazepam and alprazolam were purchased from Cambrex Company (Italy). Morphine, codeine, methadone and tramadol were prepared under the license of the Ministry of Health and Medical Education, Iran from TEMAD Company, Iran (producer of active pharmaceutical ingredients). Methamphetamine hydrochloride (HCl) and nicotine were purchased from Lipomed Pharmaceutical (Arlesheim, Switzerland). Air and nitrogen gases (99.99 % purity) were supplied by Roham Co.,Tehran, Iran.

### Method details

The research is a cross-sectional study of 616 samples completely selected from alcoholic beverages referred to the forensic toxicology laboratory of the Forensic Medicine Organization, Markazi province, Iran (March 2022 till March 2023). It should be stated that there is ban and restriction for alcoholic beverages production in Iran. All Samples have been seized from smugglers and distributors in black drug market. Forensic toxicology laboratories are affiliated to the jurisdiction authorities in all provinces in Iran. All the samples have been sent following a complaint or the judge's order for analysing alcoholic beverages in Markazi province, Iran.Quantitative methods had been validated previously for the detection of ethanol, other volatile analytes and also APIs used as a routine analytical method according to the Standard Operating Procedure (SOP) manual in the laboratory. However, some parameters such as LOD, LOQ and linearity were estimated for each analyte of interest.

Ethanol, methanol, acetone and isopropanol were analysed in all samples. Analysis was accomplished using gas chromatography with flame ionization detection (GC-FID) for the qualitative and quantitative detection of ethanol, methanol, acetone and isopropanol for forensic toxicology analysis purposes.

Routine laboratory practice requires fast and reliable and precise results. Most of the samples that are referred to forensic toxicology laboratories contain unknown amounts of alcohols, so the calibration curve has to be prepared in a wide range of concentrations to cover all concentrations. The six-point calibration curve provides the possibility to determine alcohol concentration in all samples. Calibration curve was prepared at ethanol, methanol, acetone and isopropanol concentrations of 3, 6, 15, 30, 60, 80 and 100 % volume/volume percentage (v/v percent or %v/v) for the linearity assessment. Also, LOD and LOQ were obtained for the abovementioned substances. Blank distilled water samples were spiked with low and decreasing concentrations of each drug analyte until signal/noise of about 3 was achieved using HPLC technique. LOQ was determined by performing six replicate analyses at each concentration level for all drug analytes (300–6000 ng/mL).

For the estimation of LOD and LOQ for volatile active ingredients, distilled water samples were spiked with low and decreasing concentrations of ethanol, methanol, isopropanol, and acetone until signal/noise of about 3 was achieved using GC instrumentation. LOQ was determined by performing six replicate analyses at each concentration level for all four volatile compounds at concentration ranges of 2–10 mg/L. Concentrations for standard solutions of ethanol, methanol, isopropanol, and acetone were prepared considering specific gravity for each of them. Table 1 shows LOD and LOQ for each drug analyte and also volatile compounds.

**Table 1:** Physical and chemical characteristics of alcoholic beverages analysed in Markazi Province, Iran (*N* = 616).

Physical and chemical characteristics of alcoholic beverages		Frequency (%)
**pH**	<3	2.6
	3.1–4	70.9
	4.1–5	20
	5.1–7	5
	>7.1	1.5
**Color**	Colorless	27.8
	Cherry-red	18
	Purple	4.5
	Pink	6.5
	Brown	43.2
**Appearance**	Transparent	56.8
	Translucent	43.2

All data were analysed using Chi-square test SPSS (version 25). Data analysis software was used for the calculation of concentrations of analytes in volume –volume percent (v/v %) according to the calibration curves.

### Sample preparation for the determination of added active pharmaceutical ingredients

Handmade alcoholic beverages may be produced in different conditions in unknown mediums and solvents containing fermented fruit pieces. Also, they contain many additives and organic substances. Therefore, it is necessary to extract drugs from the complex mediums using extraction techniques to remove interfering substances that can affect experimental outcomes. Dispersive liquid microextraction (DLLME) was performed as sample preparation technique to extract suspicious drugs in alcoholic beverages. For the detection of psychotropic APIs added to alcoholic drinks, two mL of alcoholic beverages were mixed with 5 mL borate buffer (pH=9) and stirred for 2 min.

A pre-prepared mixture of extraction (chloroform, 300 µL) and dispersion (methanol, 2 mL) solvents were pushed by force into the sample. The mixture was vortexed and converted to a cloudy solution for better dispersion of the organic phase into the aqueous medium. Centrifugation was performed for 5 min at 3000 rpm. The extraction solvent was sedimented after centrifugation. The extraction product containing drugs was withdrawn from the bottom of the conical tube and evaporated to dryness under a nitrogen stream. Residues were analysed as follows: Methanol (25 µL) was added to residues and, after mixing, the sample was injected into HPLC equipped with photodiode array (PDA) detector. All of the samples were analysed quantitatively.

Physical and chemical characteristics of samples including: appearance, odor, color, pH (pH was determined by the pH meter, Metrohm, Switzerland) had been inspected.

### Real samples analysis

Physical and chemical characteristics of samples including: appearance, odor, color, pH (pH was determined by the pH meter, Metrohm, Switzerland) had been inspected. All samples were prepared prior to analysis, 0.4 µL of each sample was diluted by the double distilled water until final volume reach to 1mL. After dilution of samples analysis was performed for the detection of alcohols using GC instrumentation.

A total of 616 handmade alcoholic drinks were analysed for the detection of added APIs using validated method. All samples were analysed for the detection of methanol, Ethanol, Isopropanol and acetone using GC instrumentation. Also, active pharmaceutical ingredients deliberately added to alcoholic drinks were analysed using validated method using HPLC method.

Dilution integrity is used for chromatographic methods such as GC and HPLC to confirm that the dilution procedure has no effect on the measured concentration. For samples containing an analyte concentration outside and above the validated concentration ranges, dilution was performed into the measurable range. For this purpose, quality control samples were prepared. Selected concentration of analyte (greater than LOQ) in analyte-free distilled water was spiked resulting in a known analyte concentration. Dilutability of a high concentrated sample into the validated range was achieved.It must be pointed out that the highest concentration of each analyte was achieved by serial dilution of the real samples to lie within the calibration curve concentration ranges.

## Results

Results of physical and chemical characteristics of samples are shown in Table 2.

**Table 2:** Limit of detection and limit of quantification for volatile and active pharmaceutical ingredients in alcoholic beverages analysed in Markazi province, Iran.

Analyte	LOQ	LOD
Ethanol	5 mg/*L* = 0.63 (v/v%)	1.8 mg/L
Methanol	5 mg/*L* = 0.63 (v/v%)	2 mg/L
Acetone	3mg/*L* = 0.38 (v/v%)	1 mg/L
Isopropanol	4 mg/*L* = 0.51 (v/v%)	1.5 mg/L
Oxazepam	740 ng/mL	240 ng/mL
Diazepam	391 ng/mL	129 ng/mL
Flurazepam	331 ng/mL	109 ng/mL
Alprazolam	430 ng/mL	151 ng/mL
Morphine	583 ng/mL	193 ng/mL
Codeine	795 ng/mL	263 ng/mL
Nicotine	382 ng/mL	108 ng/mL
Methamphetamine	850 ng/mL	291 ng/mL
Methadone	399 ng/mL	132 ng/mL
Tramadol	317 ng/mL	105 ng/mL

### Results of LOD, LOQ and calibration curve for each analyte

Limits were obtained for each anlysed APIs and volatile ingredients (Table 3). Linearity was obtained with the correlation coefficients R^2^>0.99, which proved good linearity for volatile analytes and also APIs detection. Calibration curve was plotted for peak area against respective concentration. Calibration curve was prepared using different concentrations of ethanol, isopropanol and acetone analysed by GC. The plot was assessed using linear regression. Linearity was obtained within the range of 300–6000 ng/mL for all drug analytes (Tables 4,5).

**Table 3:** Linearity parameters and correlation coefficients R^2^>0.99 of validated method for volatile analytes in alcoholic beverages analysed in Markazi province, Iran.

Analyte	Regression Line	R²
Methanol	*y* = 213.19x - 523.63	0.9977
Ethanol	*y* = 394.53x - 407	0.9997
Acetone	*y* = 2229.9x - 1145.1	0.9991
Isopropanol	*y* = 1048.2x - 1565.3	0.9998

**Table 4:** Linearity parameters and correlation coefficients R^2^>0.99 of validated method for psychotropic active ingredients in alcoholic beverages analysed in Markazi province, Iran (300–6000 ng/mL).

Analyte	Regression Line	R^2^
Diazepam	*y* = 1984.8x + 34,191	0.9979
Oxazepam	*y* = 392.9x + 6574	0.9924
Flurazepam	*y* = 15665x + 19,434	0.9985
Alprazolam	*y* = 10366x + 74.833	0.9971
Nicotine	*y* = 7671x + 47,839	0.9985
Codeine	*y* = 6107.9x + 150,811	0.9912
Morphine	*y* = 5154.9x + 85,959	0.9952
Methamphetamine	*y* = 2782.2x + 5399.2	0.9988
Tramadol	*y* = 10068x + 40,739	0.9986
Methadone	*y* = 868.09x - 2264.9	0.9978

**Table 5:** Concentration ranges of active pharmaceutical ingredients in alcoholic beverages analysed in Markazi Province, Iran (*N* = 616).

Analyte	Concentration (mg/dL)	Number	Total number of each drug
Morphine	1–5	4	5 (5.8 %)
	15–20	1	
Codeine	5–10	1	7 (8 %)
	11–15	4	
	16–20	2	
Methadone	3–8	6	21 (24.1 %)
	9–13	8	
	14–18	6	
	19–23	1	
Tramadol	3–7	3	12 (13.8 %)
	8–11	6	
	12–15	2	
	>15	1	
Methamphetamine	4–7	1	4 (4.6 %)
	8–10	2	
	11–13	1	
Nicotine	4–6	2	6 (6.9 %)
	7–10	2	
	11–14	2	
Diazepam	3–6	2	14 (16.1 %)
	7–9	7	
	10–12	5	
Oxazepam	5–10	2	4 (4.6 %)
	11–5	2	
Flurazepam	1–5	1	8 (9.2 %)
	6–10	5	
	11–15	2	
Alprazolam	1–3	1	6 (6.9 %)
	4–6	3	
	7–9	2	
Total		**87**	**87 (100 %)**

**Fig. 1:**
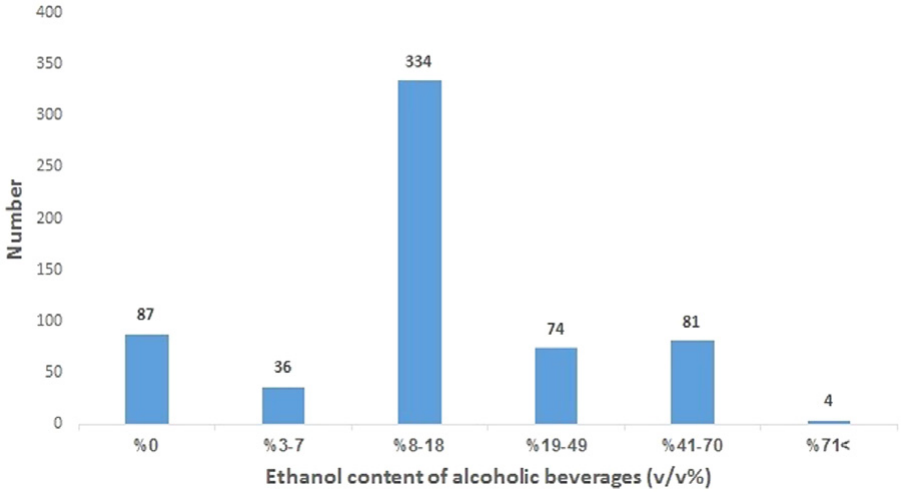
Ethanol content of handmade alcoholic beverages referred to the forensic toxicology laboratory, Forensic Medicine Organization, Markazi province, Iran.

**Fig. 2:**
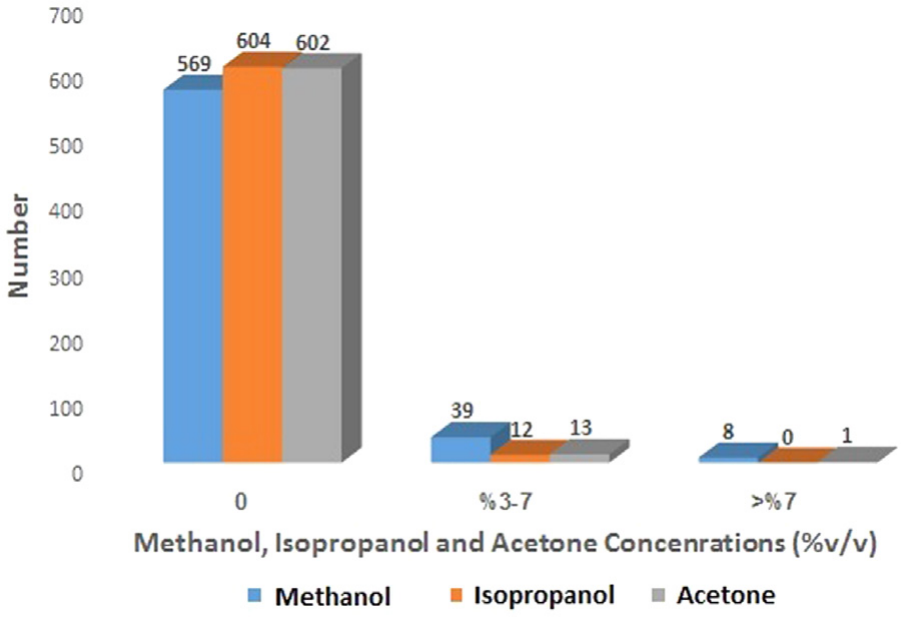
Methanol, isopropanol and acetone content of handmade alcoholic beverages referred to the forensic toxicology laboratory, Forensic Medicine Organization, Markazi province, Iran.

Results of real samples analysis showed that alcoholic beverages were adulterated with different drug categories. Table 6 shows the quantitative analysis of samples for the detection of added drugs. Figs. 1,2 show ethanol, methanol, isopropanol and acetone concentration ranges in samples.

**Table 6:** Concentration ranges of active pharmaceutical ingredients in alcoholic beverages analysed in Markazi Province, Iran (*N* = 616). Dilutability of a high concentrated sample into the validated range was achieved.

Analyte	Concentration (mg/dL)	Number	Total number of each drug
Morphine	1–5	4	5 (5.8 %)
	15–20	1	
Codeine	5–10	1	7 (8 %)
	11–15	4	
	16–20	2	
Methadone	3–8	6	21 (24.1 %)
	9–13	8	
	14–18	6	
	19–23	1	
Tramadol	3–7	3	12 (13.8 %)
	8–11	6	
	12–15	2	
	>15	1	
Methamphetamine	4–7	1	4 (4.6 %)
	8–10	2	
	11–13	1	
Nicotine	4–6	2	6 (6.9 %)
	7–10	2	
	11–14	2	
Diazepam	3–6	2	14 (16.1 %)
	7–9	7	
	10–12	5	
Oxazepam	5–10	2	4 (4.6 %)
	11–5	2	
Flurazepam	1–5	1	8 (9.2 %)
	6–10	5	
	11–15	2	
Alprazolam	1–3	1	6 (6.9 %)
	4–6	3	
	7–9	2	
Total		**87**	**87 (100 %)**

**Fig. 3:**
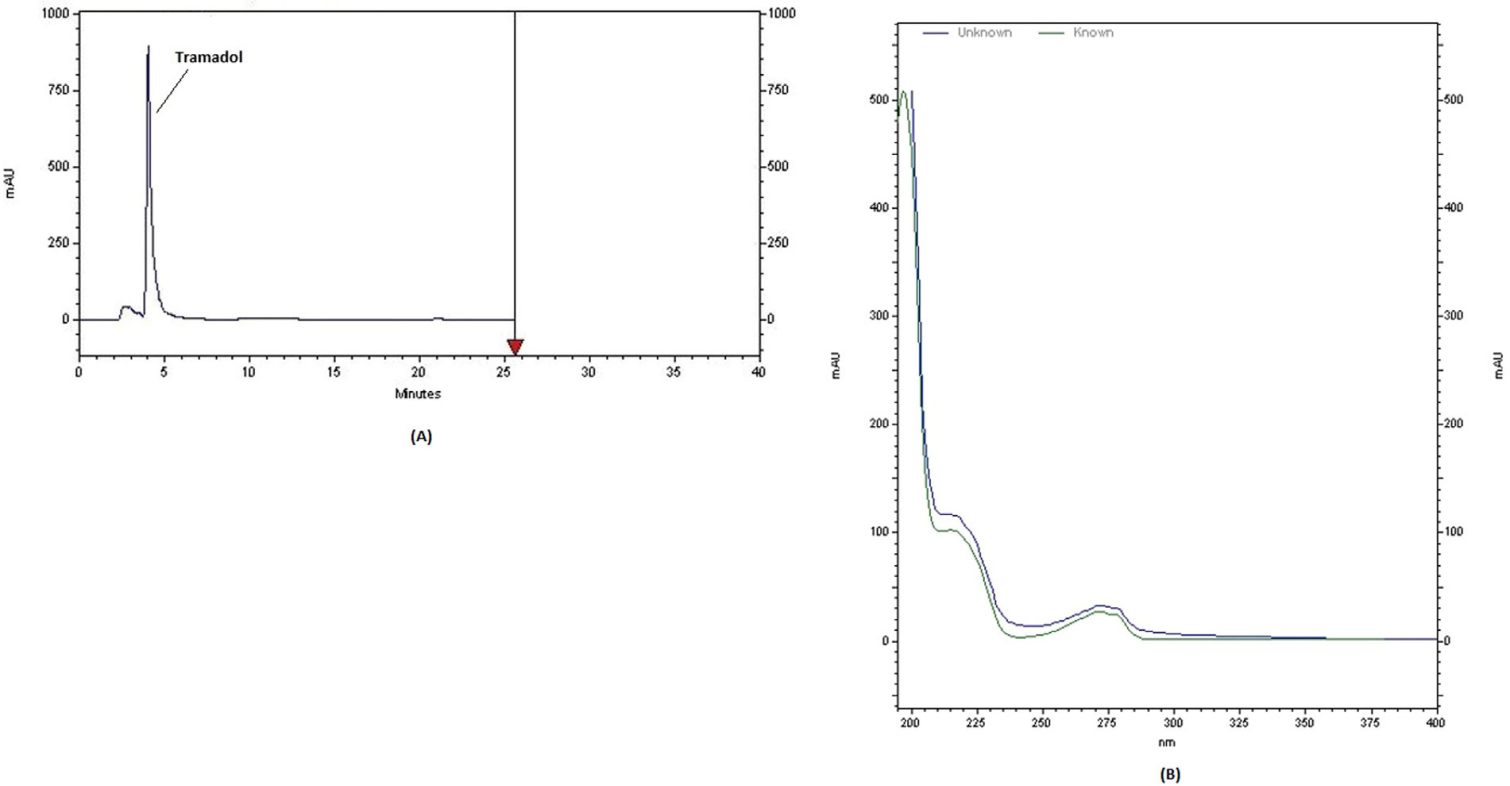
(A) Chromatogram and (B) UV spectrum of tramadol extracted from a handmade alcoholic beverage analysed in forensic toxicology laboratory, Forensic Medicine Organization, Markazi province, Iran.

**Fig. 4:**
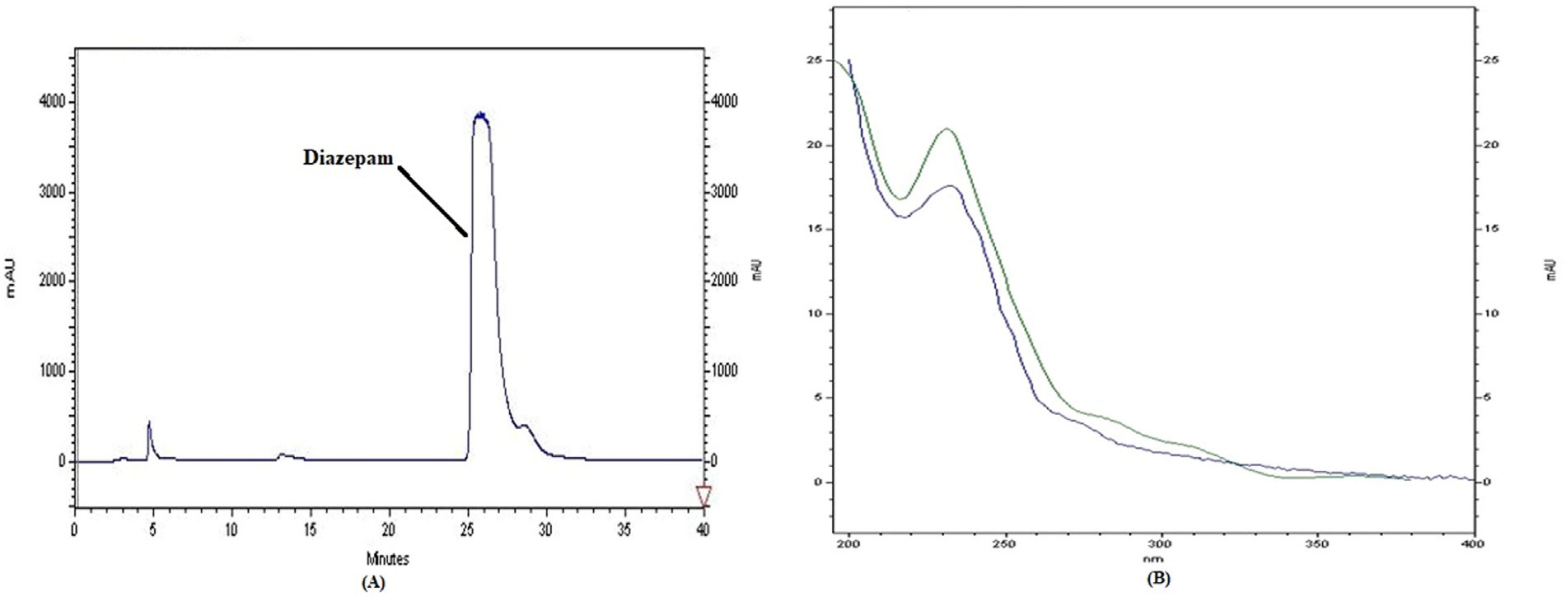
(A) Chromatogram and (B) UV spectrum of diazepam extracted from a handmade alcoholic beverage analysed in forensic toxicology laboratory, Forensic Medicine Organization, Markazi province, Iran.

**Fig. 5:**
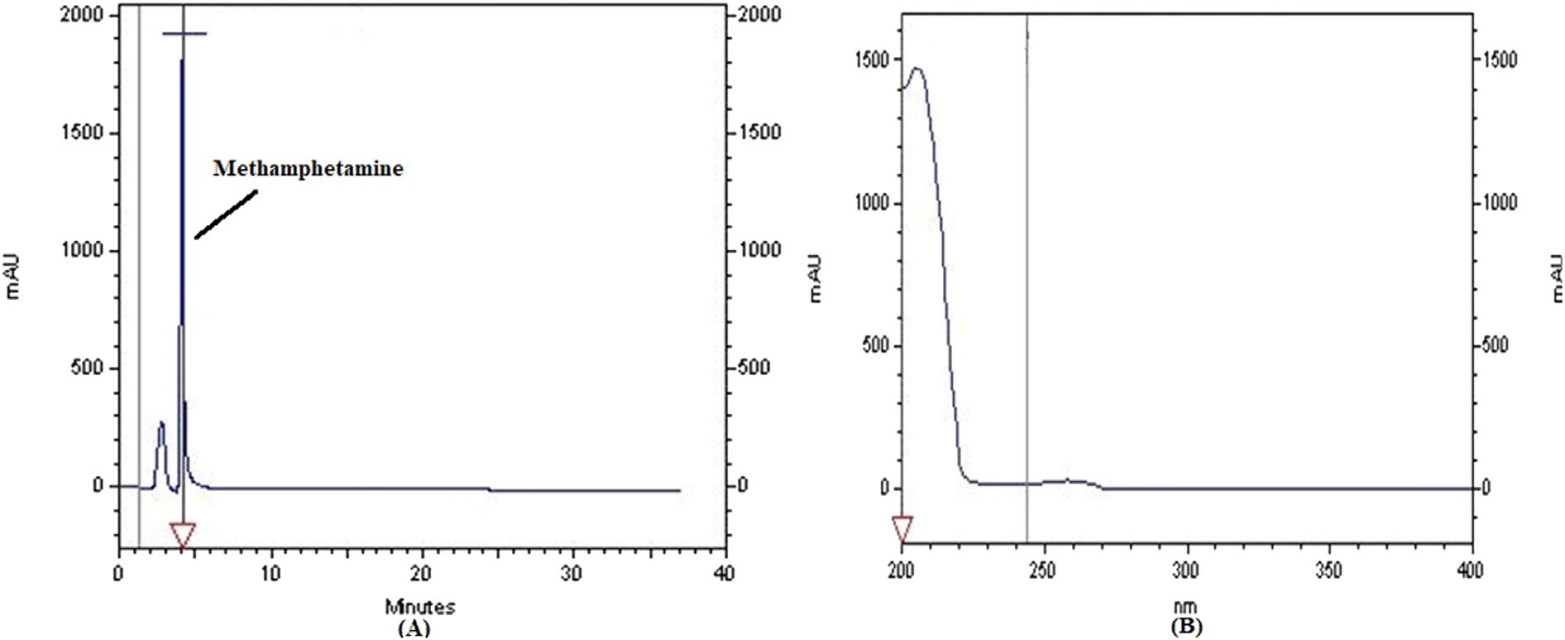
(A) Chromatogram and (B) UV spectrum of methamphetamine extracted from a handmade alcoholic beverage analysed in forensic toxicology laboratory, Forensic Medicine Organization, Markazi province, Iran.

**Fig. 6:**
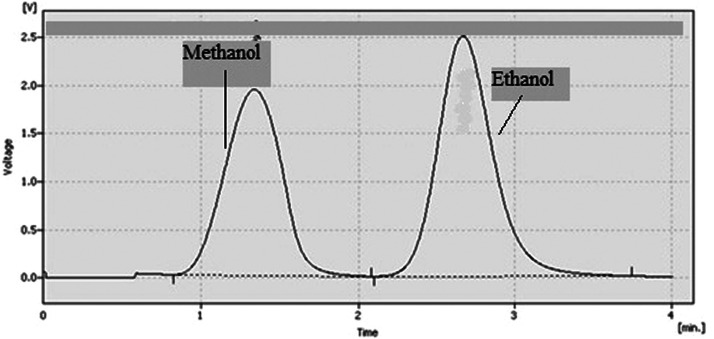
Gas chromatography chromatogram of a sample containing methanol and ethanol detected in a handmade alcoholic beverage analysed in forensic toxicology laboratory, Forensic Medicine Organization, Markazi province, Iran.

The representativeness of drug detection was evaluated by analyzing real alcoholic drink samples. All samples were analyzed using a validated method for the efficient extraction and detection of psychoactive drugs using HPLC instrumentation. Tramadol, diazepam and methamphetamine chromatogram and spectrum are shown in Fig. 3, Fig. 4, Fig. 5. Fig. 6 shows the GC chromatogram of a sample containing methanol and ethanol.

No interfering peaks were observed at the chromatographic retention times of the analytes in control matrices, indicating that the method is selective enough to be used in routine procedures.

## Method validation

### Limit of detection (LOD), limit of quantification (LOQ) and linearity assessment

Limit of detection (LOD), limit of quantification (LOQ) and linearity were assessed prior to real samples analysis. Selectivity was assured by analyzing blank samples (distilled water) spiked with the standard prevalent psychotropic drugs used in Iranian black drug market including benzodiazepines (diazepam, flurazepam, oxazepam, and alprazolam), opiates (methadone, tramadol, morphine and codeine), central nervous system (CNS) stimulants (methamphetamine and nicotine) in different concentrations. Extraction procedure was performed using dispersive liquid microextraction (DLLME) and subsequent analysis by HPLC.

Stock standard solutions of active ingredients were prepared separately in methanol at concentrations of 1 mg/mL. Standard and quality control samples were freshly prepared by appropriate dilution of stock solutions. All stock solutions were stored at −20 °C. Extraction procedure was performed to extract added drugs.

Calibration curves were obtained to assess the linearity of the method for each analyte. Blank distilled water samples were used to prepare different concentrations (300, 800, 1500, 3000 and 6000 ng/mL) of all analytes separately considering LOQ for each analyte. Plots of peak area versus concentration were made, and the relationships were determined by linear regression. The least-square method was used for the regression line preparation and expressed as correlation coefficient (R^2^).

The LOD was defined as the lower concentration of each analyte spiking in the blank distilled water that showed acceptable accuracy and precision (<20 %) analyzed in triplicate against calibration curve concentrations. All experiments (extraction procedure and instrumental analysis) were performed under the same conditions. LOD was evaluated as the concentration with a signal/noise of 3. LOQ was calculated as the concentration of analyte with a signal/noise of 10.

## Discussion

Quantitative analysis of ethanol and methanol concentrations in alcoholic beverages remains an occasional request to forensic laboratories. In forensic toxicology analyses, the need occasionally arises to determine the concentration of alcohols in seized beverages to evaluate whether a beverage is alcoholic or non-alcoholic. There are many requests from jurisdiction authorities to analyse samples for the detection of drugs as adulterants in addition to ethanol and methanol. The purpose of the present study was to analyse seized alcoholic beverages to detect psychotropic active pharmaceutical ingredients and also volatile substances in a quantitative manner. In this way, we analysed 616 seized alcoholic beverages in Markazi province, Iran.

Analysed samples showed a wide variation of color, pH, odor and also ethanol content. As alcoholic beverages are produced in non-standardized production environment using different sources of raw materials (from dilution of factory-made ethanol to fermentation of barley, grape and other fruits), they are not unique in organoleptic properties [13]. It is evident that most of the analysed samples were fermented alcoholic drinks. >90 % of analysed alcoholic drinks had the pH=3–5 in the present study. Most of the yeasts produce alcohol in the pH range similar to that is obtained in the present study [14]. This result agrees with those of earlier investigation for the analysis of homemade alcoholic beverages. They denoted that some acids such as lactic acid, produced in fermentation procedure, lowers the pH of alcoholic drinks [13].

Results of the study demonstrated that about 86 % (529) of analysed samples contained ethanol, whereas methanol was detected in 7.7 % of samples at concentration ranges 1–8 % v/v. It is important to note that the ethanol content showed substantial variation, ranging from 3 % to 71 % v/v in analysed samples. Results of the present study support those of previous works. Ghadirzadeh et al. [5] in a cross-sectional study on 100 alcoholic drinks in Tehran, Iran found ethanol in 95 % of analysed samples at concentration range 1–83 % v/v. The concentration of ethanol in >54 % of ethanol containing samples was in the range of 8–18 % in the present study. Tesfaw et al. [14] in their study on optimization of ethanol production using yeasts concluded that yeasts viability for ethanol production are influenced by ethanol concentration in fermentation medium. They also revealed that ethanol concentration >15 % may decrease the ability of yeasts for ethanol production.

In Iran, methanol poisoning continues to be a serious health issue and almost all methanol toxicities and fatalities are related to consumption of illegal and non-commercial alcoholic beverages [4,10,15]. As there is ban for all activities related to alcoholic drinks in Iran, handmade, illegally produced and smuggled alcoholic drinks are used by consumers.

Methanol may be used as a substitute for ethanol in situations where there is restriction to access to standard and factory-made ethanol containing beverages [16]. One reason for adding methanol to alcoholic drinks is to increase their ``bite'' [17]. Fruits can be used in traditional fermentation for ethanol production. Accordingly, fermentation is typically carried out by mixed cultures. Therefore, contamination with bacteria, fungi, and other yeasts could lead to the production of methanol and other products during fermentation process. Additionally, methanol is produced from pectin in the fruits. Many strains of bacteria and yeasts possess pectinolytic enzymes such as pectin methyl esterase which ultimately lead to methanol production as a result of breakdown of methoxy groups (–OCH_3_) on the galacturonic acid chain in fruit pectin [18].

During COVID-19 pandemic period, Iran struggled with one of the most serious methanol poisoning outbreaks [4,10,15]. Methanol poisoning outbreak were recorded in Turkey too [16]. Alhusein et al. (2024) recorded chemical management of two outbreaks of methanol poisoning in Riyadh, Saudi Arabia [19].

There has been an obvious rise in the prevalence of methanol contaminated alcoholic drinks since COVID-19 [10]. Defining maximum ``acceptable'' methanol content of ethanol-containing alcoholic beverages is a challenging issue. European Union limit for naturally occurring methanol in 40 % ethanol containing products is 0.4 % v/v [20]. For a 70 kg person the ``tolerable'' blood methanol concentration that is not associated with illness and do not require medical treatment is 5 mg/dL. If a 70 kg person consumes 100 mL of methanol containing drink, the corresponding ``safe'' methanol concentration would be 2 % [20]. Unfortunately, about 8 % of analysed samples in the present study contained 1–8 % v/v methanol that is far from ``acceptable'' and ``tolerable'' methanol level in alcoholic drinks. However, Dadpour et al. [6] in their analysis of alcoholic drinks found that methanol was detected in <0.5 % of the seized samples analyzed in Mashhad city, Iran. The low price and availability of methanol is considered as one of the significant factors in detecting methanol in alcoholic drinks [15].

Depending on the preparation (fermentation or distillation) and purification methods, alcoholic beverages have a varying percentage of ethanol or other toxic alcohols such as methanol [21]. Distillation of fermented alcoholic drinks increases ethanol concentration. Isopropanol and acetone were detected in 12 and 14 samples respectively. Alcohols with more than two carbon atoms known as higher alcohols, (including isopropanol) are by products of fermentation process [22]. Isopropanol consumption can cause respiratory and CNS depression [4]. The source of acetone in alcoholic beverages is not clear. It may be added deliberately to alcoholic drinks. One assumption is the conversion of isopropanol to acetone during fermentation procedure. Despite obtained results, Dadpour et al. [6] had not detected acetone in alcoholic drinks. Hexanoic acid, phenethyl alcohol and other fermentation byproducts were detected in the study of Davis, Hickey and Goodpaster [8]. One of the important obtained results in the present study was the detection of APIs in 87 (14.12 %) of analysed samples, in different concentrations. Alcoholic drinks are produced in non-standard clandestine laboratories without any regulation and control. Davis, Hickey and Goodpaster [8] validated a method for the detection of ɣ-hydroxybutyric acid (GHB) and ɣ-butyrolactone (GBL) in alcoholic beverages via total vaporization solid-phase microextraction (TV-SPME) and gas chromatography–mass spectrometry. They found GHB and GBL in beer, wine, rum and cola severed in night clubs, parties and raves. One possible explanation for obtained results is to increase psychoactive properties which are due to the pharmacologic effects of added drugs. Willingness to consume alcohol to the point of euphoria encourages alcohol users to buy alcoholic drinks with more psychoactive effects from fixed dealers. GHB and GBL may be spiked into alcoholic beverages at parties, raves, or night clubs without the victim's knowledge [8]. Although benzodiazepines, opioid drugs such as methadone and tramadol are categorized as under control drugs, they are available and can be obtained without any prescription ( [3]. Therefore, free access to controlled drugs and substances and leakage of drugs from legitimate centers to drug grey/black market, drug diversion from medical therapy to non-medical use and selling the medication by drug dealer are among the reasons that increases drug use in alcoholic drinks [23,24].

In line with the obtained results in the present study, Papaspyridakou, Giannoutsou and Orkoula [25] found limit of quantification for ethanol, methanol and isopropanol as 0.5 %. 0.39 % and 0.16 % v/v respectively.

It is important to emphasize that APIs are deliberately added to alcoholic drinks and none of them are produced during dilution or fermentation process. Ghadirzadeh et al. [5] analysed 100 alcoholic beverages for quantitative analysis of ethanol and qualitative determination of methadone, tramadol and benzodiazepines. However, the results are in disagreement with those of Ghadirzadeh et al's. (2019) study, in that, they found tramadol as the most prevalent API. But methadone was detected as the most common APIs in this study. There are some reasons for this discrepancy, difference between geographic locations of the study is an important factor to get different results. The other reason may be attributed to the change of trend and pattern of drug use and availability over time.

Oral pharmaceutical dosage forms of methadone are 25 mg/5 mL, 5 mg/5 mL and 25 mg/mL solution and syrup. Solid dosage forms of methadone are 5, 20 and 40 mg tablets in Iran. Although methadone concentration in alcoholic beverages is less than pharmaceutical dosage forms, but drug-drug interaction between ethanol, methadone and other CNS depressants such as tramadol and benzodiazepines would be a harm for consumers.

Tramadol is available in Iran as 50, 100 mg tablets and capsule and 50 mg/mL ampule. These dosage forms can be easily dissolved in alcoholic drinks. If one 100 mg tablet is dissolved in one liter of alcoholic drink, it makes a concentration equal to 0.1 mg/mL that is close to the concentration ranges for tramadol in analysed samples. Although the concentration of tramadol is not more than recommended therapeutic dose, but concomitant non-medical use of opioid agonists and other CNS depressants with alcohol would have synergistic effects and cause respiratory and CNS depression and even death [23,26]. Four samples in the present study contained methamphetamine. Methamphetamine may be added to drinks to induce euphoria. There is a misconception that methamphetamine reduces poor sexual activity, loss of energy, and attention that are related to methadone use. Methamphetamine abuse is accompanied by an array of health consequences. This controlled substance is widely available and used in Iran, causing death in young and middle age population [27].

In contrast with the results obtained by Tsenang et al. [13] that analysed chemical constituents in handmade morula beers with no harmful substances, results of the present study verified that methadone, diazepam and tramadol were the most prevalent APIs detected in alcoholic drinks. Methadone and tramadol are widely abused in Iran. Previous reports concerning deaths due to the use of opioid drugs such as methadone and tramadol in single or multidrug use pattern confirm their illegal use [23].

Diazepam was the most detected benzodiazepine in analysed samples. Diazepam, Oxazepam, flurazepam and alprazolam were detected in 32 samples, which has also been observed in the previous study [5]. Benzodiazepines are classified as controlled drugs by the Ministry of Health and Medical Education, Iran. But they are freely available. Use of benzodiazepines in beverages may be due to their CNS depressive effects. Obtained results in the present study are in agreement with those of Rao et al. [9] They developed an instrumental HPLC method for the detection of alprazolam, chloral hydrate and diazepam in traditional alcoholic beverages. About 60 % of analysed samples in their study were adulterated with APIs. Teoh et al. [28] determined three sedative-hypnotics (ketamine, nimetazepam, and xylazine) from drug-spiked beverages using a vortex-assisted dispersive liquid–liquid microextraction-gas chromatography (VADLLME-GC). LOD for three detected drugs were in the range that was obtained for APIs in the present study.

Morphine and codeine were two opium alkaloids that were detected in alcoholic drinks. One assumption for their detection is that crude opium was dissolved in the beverage.

It is important to emphasize that the present study has some limitations. Only a few active pharmaceutical ingredients were analysed in seized alcoholic beverages quantitatively. However, as a result of using prevalidated methods in the laboratory, it is worth mentioning that no other active ingredients were detected in the process of analyzing samples using DLLME as sample preparation and HPLC as analytical technique.

The strengths of this study include the use of high-quality survey data with representative samples from a well-established study conducted in Markazi province, Iran. Further studies are required to investigate handmade alcoholic beverages in other provinces in Iran and make a warning network for health authorities and also consumers.

## Conclusion

Results revealed that adulterated alcoholic beverages may contain different amounts of psychoactive substances. There are circumstances in which drinks are adulterated with active pharmaceutical ingredients or toxic alcohols other than ethanol, to increase the pharmacologic or desired effects and also lower their cost. Such adulterations may cause poisonings and mortality of those affected. Analysis results can be useful for safety evaluation, adulteration detection, provenance and differentiation between different kinds of alcoholic beverages.

## Limitations

It is important to emphasize that the present study has some limitations. Only a few active pharmaceutical ingredients were analysed in seized alcoholic beverages quantitatively.

## Ethics statements

This study was done under the supervision of the Legal Medicine Research Center, Legal Medicine Organization, Tehran, Iran and the protocol was approved by the Islamic Azad Tehran Medical Sciences University, Pharmacy and Pharmaceutical Branches Faculty which is registered under the registration number IR.IAU.PS.REC.1399.297.

## CRediT author statement

**Morvarid Sadat Miri:** Methodology, Analysis, Validation, Writing. **Dariush Badakhshan:** Methodology, Data curation, Supervision, Analysis, Validation. **Maryam Akhgari:** Methodology, Validation, Analysis, Writing – review and editing. **Masoud Ghadipasha:** Methodology, Data curation, Supervision, Analysis, Validation.

## Declaration of competing interest

The authors declare that they have no known competing financial interests or personal relationships that could have appeared to influence the work reported in this paper.

## Data Availability

Data will be made available on request.
